# TRAF6 and IRF7 Control HIV Replication in Macrophages

**DOI:** 10.1371/journal.pone.0028125

**Published:** 2011-11-28

**Authors:** Mélissa Sirois, Lynda Robitaille, Robin Allary, Mohak Shah, Christopher H. Woelk, Jérôme Estaquier, Jacques Corbeil

**Affiliations:** 1 Department of Molecular Medicine, Infectious Disease Research Center, CHUL Research Center and Laval University, Québec, Québec, Canada; 2 Department of Electrical and Computer Engineering, Centre for Intelligent Machines, McGill University, Montreal, Québec, Canada; 3 Department of Medicine, University of California San Diego, San Diego, California, United States of America; 4 CNRS FRE3235, Université René Descartes Paris, Paris, France; INSERM, France

## Abstract

The innate immune system recognizes virus infection and evokes antiviral responses which include producing type I interferons (IFNs). The induction of IFN provides a crucial mechanism of antiviral defense by upregulating interferon-stimulated genes (ISGs) that restrict viral replication. ISGs inhibit the replication of many viruses by acting at different steps of their viral cycle. Specifically, IFN treatment prior to *in vitro* human immunodeficiency virus (HIV) infection stops or significantly delays HIV-1 production indicating that potent inhibitory factors are generated. We report that HIV-1 infection of primary human macrophages decreases tumor necrosis factor receptor-associated factor 6 (TRAF6) and virus-induced signaling adaptor (VISA) expression, which are both components of the IFN signaling pathway controlling viral replication. Knocking down the expression of *TRAF6* in macrophages increased HIV-1 replication and augmented the expression of IRF7 but not IRF3. Suppressing *VISA* had no impact on viral replication. Overexpression of IRF7 resulted in enhanced viral replication while knocking down *IRF7* expression in macrophages significantly reduced viral output. These findings are the first demonstration that TRAF6 can regulate HIV-1 production and furthermore that expression of IRF7 promotes HIV-1 replication.

## Introduction

Infection by RNA viruses, such as HIV-1, initiates antiviral innate immune responses by inducing type I IFNs [1], [2]. The treatment of primary human macrophages *in vitro* with type I IFN prior to HIV-1 infection inhibits virus replication, indicating that potent inhibitory factors are present [3], [4]. However, pre-treatment is not a viable clinical option and, ultimately, innate immune responses *in vivo* fail to completely protect the human host even though genes integral to host defense are expressed. This may be due to the deregulation by HIV-1 of the signaling events necessary for induction of an appropriate innate immune response mediated by IFN or that HIV-1 replication outpaces these defenses. Type I IFNs display diverse biological effects that restrict virus replication by upregulating the expression of numerous genes (ISGs) [5]–[13]. For example, *eukaryotic translation initiation factor 2-alpha kinase* (*EIF2AK2*, also known as *PKR*), *oligoadenylate synthetase 1 (OAS1)* and *interferon-stimulated gene 15 (ISG15)* are known to be anti-HIV ISGs [14]–[16]. However, HIV-1 circumvents the protective effects of IFN and may even upregulate certain ISGs to its benefit [17]. Recently, Smith and collaborators identified ISGs expressed in inguinal lymph nodes that were positively associated with HIV-1 viral replication [18]. Moreover, it has been shown that the level of Type I IFN correlates with AIDS pathogenesis [19].

Production of IFN is induced by two major receptor systems for detecting RNA viruses: the toll-like receptors (TLRs) and cytoplasmic retinoic acid-inducible gene I (RIG-I)-like helicases (RLHs) [20], [21]. The adaptor molecule TRAF6 has been shown to be involved in the TLR signaling pathway and activates IRF7, IRF3 and nuclear factor kappa B (NFkB) [22], [23]. Furthermore, the RLH signaling pathway involves RNA helicase RIG-I and melanoma differentiation associated protein-5 (MDA5) [24], [25] that interact with VISA through a caspase recruitment domain (CARD/CARD) interaction [26]–[29]. VISA induces I-kappaB kinase (IKKε and TANK binding kinase 1 (TBK1) which are responsible for the activation of IFN-regulatory factors (IRF3 and IRF7) through phosphorylation and consequently the production of type 1 IFN [30], [31]. IFN production is cell type and stimuli specific and our aim was to ascertain the phenotype in the process of HIV-1 infection of primary human macrophages.

The goal of this study was to identify factors belonging to the interferon pathway that are altered during HIV-1 infection which contribute to the modulation of viral replication. We identified genes involved in the IFN signaling pathway that were impacted by HIV-1 infection of human primary macrophages. Our model is highly relevant to HIV-1 infection since macrophages are among the first cell types infected during transmission of HIV-1. In addition, macrophages infection by SIV is similar to that of CD4+ T cells in the acute phase of SIV infection [32]. We demonstrated that IFNα2 pre-treated macrophages, infected or not with HIV-1, modulated genes that were involved in the transcriptional regulation of the IFN pathway. Among them, HIV-1 downregulated TRAF6 and VISA gene expression. The downregulation of TRAF6 in macrophages infected with HIV-1 resulted in enhanced viral replication. Suppression of TRAF6 resulted in increased expression of IRF7. Overexpression of IRF7 lead to enhanced viral expression and suppression of IRF7 resulted in diminished viral output. The characterization of antiviral innate immunity genes modulated by HIV-1 infection provides a greater understanding of the mechanisms that may be used to combat the virus and improve antiviral treatments.

## Results

### Type I IFNα2 inhibits HIV-1 replication in primary macrophages

**Figure 1:**
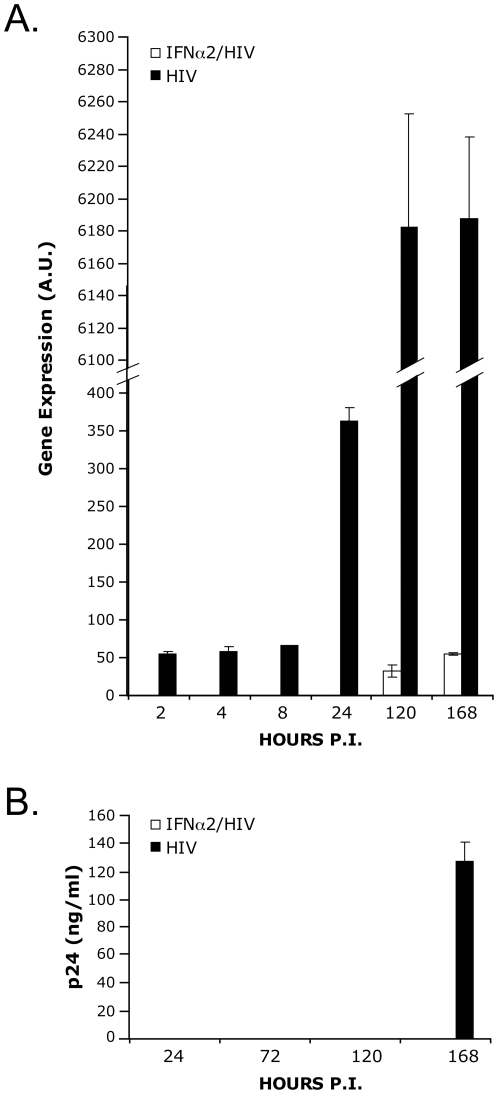
Type I IFNα2 inhibits HIV-1 replication in primary macrophages. A) Gene expression of *TAT spliced* at 2, 4, 8, 24, 120 and 168 hours post-infection of macrophages. The macrophages were incubated in the absence or presence of IFNα2 (1000 UI/ml for 18 hours). The expression level was calculated by qRT-PCR assessments of triplicates and normalized to the level of 18 S. Results shown are a representative of 2 independent experiments. A.U. corresponds to arbitrary units. B) Cell-free culture supernatants were collected at 24, 72, 120 and 168 hours post-infection and analyzed for p24 content. Results shown represent 2 combined independent experiments.

Kornbluth and collaborators demonstrated that macrophages treated 18 hours with 1000 IU/ml of IFNα2 prior to HIV-1 NL4-3BaL*env* strain infection inhibited viral production [3]. To ascertain the stage at which HIV-1 replication was curtailed in the IFNα2 pre-treated macrophages, we monitored viral replication by quantifying *TAT spliced* message expression by qRT-PCR, a marker of productive viral transcription distinct from incoming viral genomic RNA. Treatment of infected cells with a non-nucleoside reverse transcriptase inhibitor, efavirenz, blocked all steps of infection after entry and prevented the appearance of *TAT spliced message*, demonstrating specificity for new viral production (data not shown). *TAT spliced* message could be detected as early as 2 hours post-infection, remained stable until 8 hours, and increased over time thereafter in macrophages infected with HIV-1. It is possible that initial *TAT spliced* detection could be due to viral nucleic content that's encapsidated within virions. Still, kinetic was greatly delayed in IFNα2 pre-treated macrophages infected with HIV-1, where initial detection was observed at 120 hours and subsequently increased over time (Fig. 1). Similarly, HIV-1 p24 antigen could be detected in cellular supernatant in untreated samples at day 7 after infection. For the purpose of this study, we used the early TAT spliced message detection since the HIV-1 p24 can only be detected from day 7 in our model.

To determine the effect of HIV-1 on the IFN response and uncover potential effectors of HIV-1 replication, gene expression analysis in HIV-infected and non-infected IFNα2-treated macrophages was compared. This strategy enabled the identification of interferon-induced genes that were differentially modulated during HIV-1 infection.

### HIV-1 modulates TRAF6 and VISA gene expression in IFNα2 pre-treated macrophages

To identify specific genes involved in Type I IFN signaling modulated by HIV-1 infection, gene expression in IFNα2 pre-treated macrophages infected or not with HIV-1 was measured at 2, 4, 8, and 24 hours post-infection using high-density oligonucleotide microarrays (U133 Plus 2.0 arrays). Identification of the most highly modulated genes was ascertained by applying the short time series analysis framework of Shah and Corbeil with a linear kernel [33]. The application of linear kernel in conjunction with the framework results in a configuration that effectively scores the genes based on averaged differences between consecutive measurements scaled by their respective means, and this, over all the time points in the two conditions tested. We then compared the results obtained to untreated and uninfected control cultures. The most significantly up or downregulated genes were then identified as the ones obtaining extreme (respectively high and low) scores. This analysis demonstrated significant differences in IFNα2 pre-treated macrophages infected with HIV-1 when compared to the IFNα2 pre-treated macrophages only. Analysis of the top 500 differentially expressed genes (maximum scores of comparison) between IFNα2 treated macrophages that were either infected or not with HIV-1 through all time points was performed using Database for Annotation, Visualization and Integrated Discovery (DAVID) [34]. This analysis identified 32 significant functions. Table 1 displays the ten most significant functional categories associated with genes differentially modulated. The same gene can be related to more than one function and the statistical significance takes account of this fact. Examination indicated that most of these functions are related to transcription. Table 2 provides a short list of known genes associated with IFN response. The expression of most known genes related to the interferon response, such as *OAS1* and *2* (2′, 5′ oligoadenylate synthetase 1 and 2), *MX1* and *2* (myxovirus (influenza virus) resistance 1 and 2*)* and IRF7 in IFNα2 pre-treated macrophages compared to untreated control were detected early and throughout the time course. Furthermore, HIV-1 antiviral factors, such as APOBEC3G, APOBEC3A and TRIM22, were upregulated in IFNα2 pre-treated macrophages compared to untreated control. However, most of these IFN-related genes were unaffected by the presence of HIV-1 after the IFNα2 treatment.

**Table 1:** Ten most significant functional categories associated with genes differentially modulated in IFNα2 pre-treated macrophages infected or not with HIV-1.

Function	p-value	No. of genes
Phosphoprotein	1,9E-08	126
Alternative splicing	9,1E-05	127
Nucleus	3,3E-04	90
RNA-binding	1,3E-03	19
DNA-binding	2,2E-03	47
Chromosomal rearrangement	3,0E-03	12
Transcription	5,4E-03	46
Transcription regulation	6,6E-03	45
Peroxidase	6,9E-03	4
Cytoskeleton	7,7E-03	14

Functional categories were obtained using the DAVID Bioinformatics tool. Selection of the ten most significant functional categories out of 32 functions involved the top 500 genes differentially modulated in IFNα2 pre-treated macrophages infected or not with HIV-1.

**Table 2:** Peak expression and time point of known genes associated in IFN response that were modulated in IFNα2 pre-treated macrophages compared to untreated control.

IFN-relatedgenes	ReferenceSequence	Peak expression(Fold induction)	Time(hours)
APOBEC3A	NM_145699	1267	2
APOBEC3G	NM_021822	3	2
EIF2AK2	NM_002759	3	4
G1P2 (ISG15)	NM_005101	47	2
G1P3	NM_002038	10	24
IFI27	NM_005532	1131	4
IFI44L	NM_006820	1182	2
IFIT2	NM_001547	74	2
IFIT3	NM_001549	32	8
IFITM1	NM_003641	707	4
IFITM3	NM_021034	27	8
IRF1	NM_002198	3	8
IRF7	NM_001572	12	2
ISG29	NM_002201	702	2
MX1	NM_002462	197	8
MX2	NM_002463	24	2
OAS1	NM_016816	33	2
OAS2	NM_016817	41	2
OASL	NM_003733	446	2
TRIM22	NM_006074	11	2

**Figure 2:**
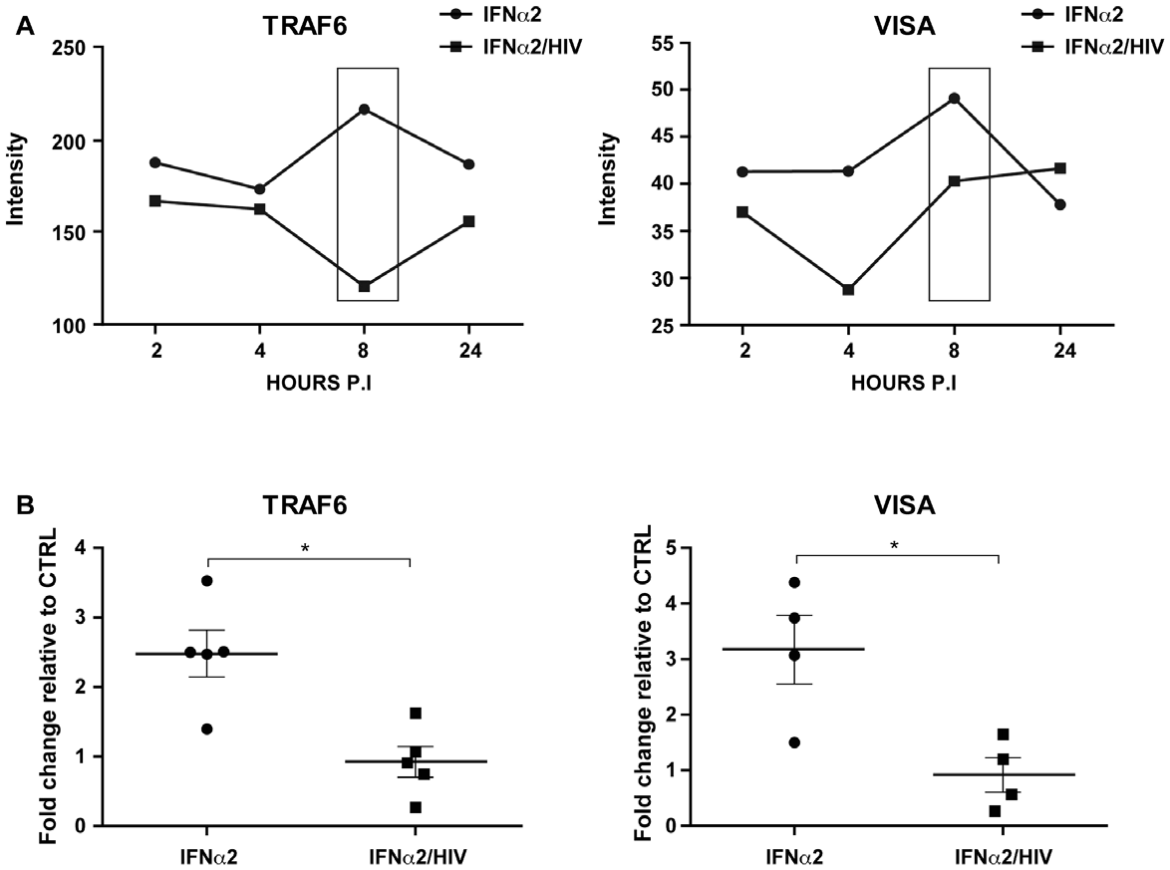
HIV-1 modulates TRAF6 and VISA expression in IFNα2 pre-treated macrophages. A) Intensity of the expression of the genes of interest obtained from the oligonucleotide array analysis (U133 Plus 2.0 array) at 2, 4, 8 and 24 hours post-infection of macrophages. Cells were treated with IFNα2 (1000 UI/ml for 18 hours) with or without HIV-1. B) Validation of the regulation of gene modulation by HIV-1 in IFNα2 pre-treated macrophages (differentiated with human serum) at 8 hours post-infection. Non-IFNα2 treated and non-HIV infected macrophages were used as negative control. An * denotes a significant difference (P<01, paired *t*-test) between IFNα2 pre-treated or not macrophages. The expression level was calculated by qRT-PCR assessments obtained from four donors and normalized to the level of 18S.

Our intent was to identify genes related to interferon that were inhibited by HIV-1. Table 3 shows candidate interferon-related genes from the top 500 genes that were modulated significantly when HIV-1 was present. These genes represent potential regulators of interferon and innate immunity pathways that are modulated by HIV-1 infection in macrophages. We were specifically interested in VISA and TRAF6 since these two factors represent key control points in the regulation of the IRFs pathway, ISGs production and innate immunity, and expression of these two genes was reduced when HIV-1 was present. Therefore, we examined *TRAF6* and *VISA* gene expression over time to assess the dynamics of their expression. As shown by the oligonucleotide array analysis, the greatest differences in gene expression for these two genes occurred at 8 hours post-infection (Fig. 2A). To validate the modulation of HIV-1 on host gene expression of the IFNα2 pre-treated macrophages, we evaluated independent donors, under the same conditions as the oligonucleotide array experiment. Altogether we used 15 independent donors to perform the experiments described (see material and methods section). We tested the expression of *TRAF6* and *VISA*, using qRT-PCR (Fig. 2B). *TRAF6* and *VISA* gene expression were upregulated in IFNα2 pre-treated macrophages but downregulated after HIV-1 infection at 8 hours. The downregulation of *TRAF6* and *VISA* after HIV-1 infection of IFNα2 pre-treated macrophages suggested a possible role for these factors in the regulation of HIV-1 expression.

**Table 3:** Peak expression and time point of candidate genes associated with IFN response that were modulated in IFNα2 pre-treated macrophages compared to untreated control and modulated by HIV-1.

IFN-relatedgenes	ReferenceSequence	Peak expressionIFNα2/CTRL(Fold induction)	Time(hours)	Peak expressionIFNα2/HIV infection(Fold induction)	Time(hours)
BIRC3	NM_001165	7	2	3,5	2
CCL5	NM_002985	8	2	4	8
CD38	NM_001775	39	4	13	16
CEBPD	NM_005195	2	8	1	16
CHI3L1	NM_001276	2	2	1	2
CXCL10	NM_001565	124	2	31	2
CXCL11	NM_005409	118	2	39	8
CXCL9	NM_002416	28	2	9	16
EHF	NM_012153	4	8	1	16
HMGA1	NM_145899	2	8	1	24
LILRB2	NM_005874	16	8	8	2
NCOA3	NM_181659	2	2	4	4
OGT	NM_181632	5	8	4	2
PIGR	NM_002644	3	8	1,5	8
PIP5K2A	NM_005028	3	8	1,5	8
SAMHD1	NM_015474	2	8	1	8
TAP2	NM_000544	16	2	8	2
TCF4	NM_003199	2	2	0,7	16
TRAF6	NM_004620	2	8	1	8
TXNIP	NM_006472	2	8	1	16
VISA	NM_020746	2	8	1	8

Selection of the top 500 most modulated genes when comparing the IFNα2 pre-treated macrophages infected or not with HIV-1. Of these, 55 were related to interferon as a keyword. From these 55, 21 were upregulated by interferon as compared to untreated control (peak expression and time point) but modulated differentially when HIV-1 was added. Those genes may represent interferon effectors that are modulated by HIV-1 infection during the interferon response.

### *TRAF6* but not *VISA* controls the level of expression of IRF7 and HIV-1 replication

**Figure 3:**
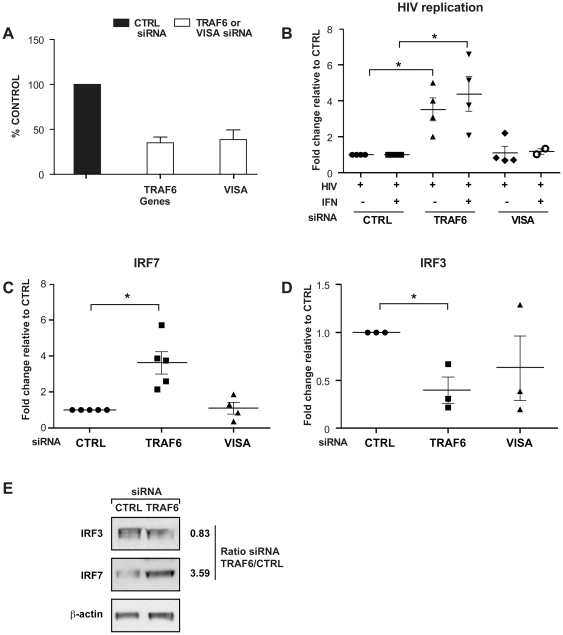
*TRAF6* but not *VISA* controls the level of expression of IRF7 and HIV replication. Primary human macrophages were subjected to siRNA knockdown for *TRAF6* and *VISA*. The concentration of siRNA used was 200 nM. A) Percentages of inhibition of *TRAF6* and *VISA* in the presence of specific siRNA targeting both gene individually [n = 3] compared to a control non-targeted siRNA [n = 3]. Level of expression was measured by qRT-PCR B) Macrophages were all infected with HIV-1 with and without an IFNα2 pretreatment, and treated with specific siRNA. The level of *TAT spliced* message measured 24 hours post-infection in TRAF6 and VISA conditions were compared to the negative control siRNA. C) Level of IRF7 and D) Level of IRF3 message expression in the absence of IFNα2 when TRAF6 and VISA are repressed by siRNA compared to the negative control siRNA. The qRT-PCR results were normalized to the level of 18S. An * denotes a significant difference (P<0.05, one sample *t*-test) compared to the negative control siRNA. Each symbol in a group represents one independent donor. E) Western blot for IRF3 and IRF7, 24 hours post-infection in macrophages with TRAF6 suppression compared to siRNA control. The densitometry of the bands was normalized to that of β-actin and shows a decrease in protein level of IRF3 and an increase of IRF7. The ratio is shown next to the respective panels. Results shown are representative of 3 independent experiments.

To analyze the impact of *TRAF6* and *VISA* in the context of HIV-1 infection, we suppressed both genes independently using siRNA. Gene expression inhibition levels of 65% and 61% were obtained for *TRAF6* and *VISA* respectively as compared to a non-targeted siRNA negative control (Fig. 3A). To further decipher the role of TRAF6 in the IFN antiviral response, we evaluated HIV-1 replication, 24 hours after infection of IFNα2 pre-treated or not macrophages. HIV-1 replication was significantly upregulated in macrophages with knockdown expression of *TRAF6* as compared to the control pre-treated macrophages independently of IFN treatment (Fig. 3B). This is the first report that TRAF6 can function to limit HIV-1 replication. No significant differences were detected in macrophages treated with *VISA* siRNA when compared to the negative control siRNA irrespective of the presence of IFN pretreatment (Fig. 3B).

In this transduction pathway, TRAF6 activates IRF3 and IRF7 to promote their transcriptional functions [22], [23], [30], [31]. Therefore, we evaluated the expression of IRF3 and IRF7 genes in this context. As shown in figure 3C and D, upregulation of HIV-1 replication in macrophages with knockdown of *TRAF6* expression seen in figure 3B is accompanied by significant overexpression of *IRF7* compared to the HIV-1 infected negative control (Fig 3C). No significant difference in the expression of *IRF7* in HIV-1 infected macrophages with knockdown of *VISA* expression was observed. Conversely, *IRF3* was downregulated in HIV-1 infected TRAF6 knockdown macrophage, while no significant difference in the expression of *IRF3* in the *VISA* siRNA treated targets was found (Fig. 3D). This suggested that VISA was not responsible for the observed increase in viral replication. It is possible that alternative effectors can substitute for its function or that its modulation has no bearing on HIV-1 production.

To validate our finding at the protein expression level, we performed western blotting for both IRF3 and IRF7. Levels of these proteins in lysates of *TRAF6* knockdown or control siRNA treated (200 nM) macrophages from three independent donors were normalized using β-actin. As seen in figure 3E, IRF3 is downregulated (0.83 fold, corresponding to a 17% reduction) and IRF7 is upregulated (3.59 fold) in *TRAF6* knockdown macrophages compared to control siRNA. The protein data confirmed our gene expression results and suggested that the regulation of HIV-1 replication by TRAF6 involved IRF7.

### IRF7 downregulation decreases HIV-1 replication

**Figure 4:**
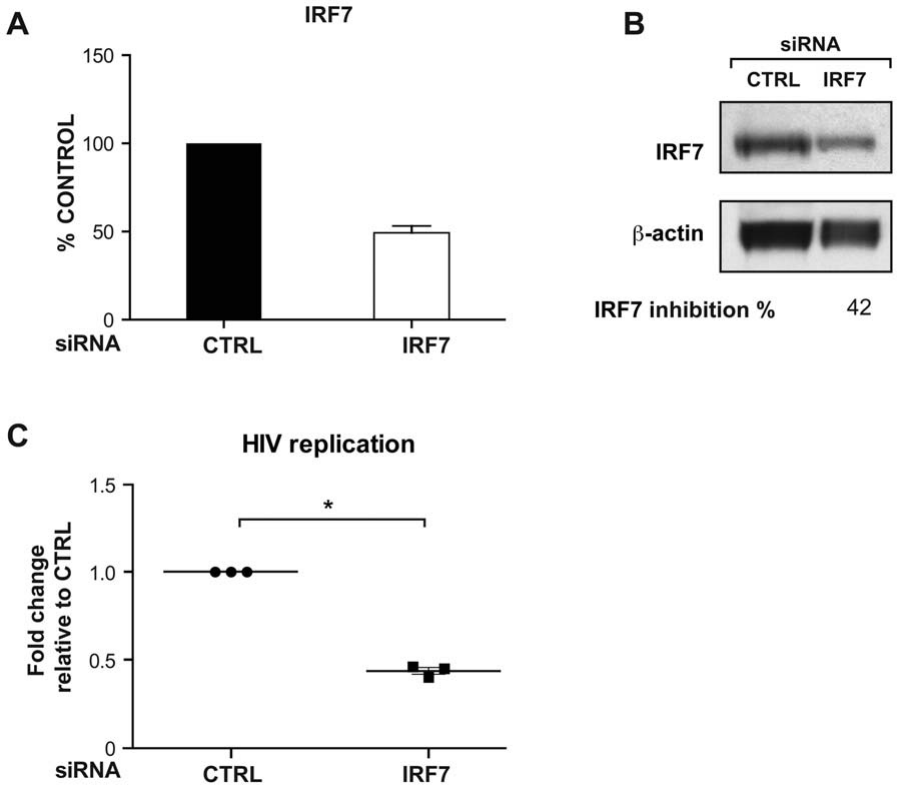
IRF7 expression modulates HIV-1 replication in primary macrophages. A) Level of *IRF7* gene expression in macrophages treated with siRNA targeting *IRF7* (50 nM) [n = 3] compared to a control non-targeted siRNA [n = 3] resulted in a 55% reduction in *IRF7* gene expression. The qRT-PCR results were normalized to the level of 18 S. B) Western blot for IRF7 protein treated as in panel A shows a decrease of 42% for IRF7 protein. The densitometry of the bands was normalized to that of β-actin. Results shown are representative of 2 independent experiments. C) Level of *TAT spliced* expression following inhibition (51%) by IRF7 as compared to control. The qRT-PCR results were normalized to the level of 18 S. An * denotes a significant difference (P<0.001, one sample *t*-test) compared to the negative control siRNA. Each symbol in a group represents one independent donor.

To elucidate if IRF7 directly alters HIV-1 replication, we first evaluated the effect of downregulation of IRF7 expression using siRNA. Macrophages were transfected with specific siRNAs targeted against *IRF7* or silencer negative control. As shown in figure 4, transfection of specific *IRF7* siRNA (50 nM) significantly reduced IRF7 mRNA (55% reduction) (Fig. 4A) and protein (42% reduction) (Fig. 4B) expression. The inhibition of IRF7 resulted in a comparable downregulation of HIV-1 replication (51% reduction) (Fig. 4C).

### IRF7 overexpression increases HIV-1 replication

**Figure 5:**
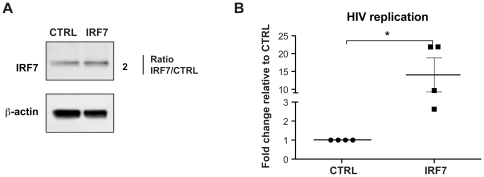
IRF7 overexpression increases HIV-1 replication in primary macrophages. A) Western blot for IRF7 protein, 24 hours post-transfection of macrophages overexpressing IRF7 showed a 2 fold increase compared to empty vector control. The densitometry of the bands was normalized to that of β-actin. The ratio is shown next to the respective panels. Results shown are representative of 2 independent experiments. B) *TAT spliced* expression was augmented 14 fold in primary human macrophages overexpressing IRF7 compared to empty vector control. The qRT-PCR results were normalized to the level of 18 S. An * denotes a significant difference (P<0.05, one sample *t*-test) compared to the empty vector control. Each symbol in a group represents one independent donor.

To confirm that IRF7 promoted HIV-1 replication, IRF7 was overexpressed in macrophages followed by infection with HIV-1 at a multiplicity of infection (MOI) of 0.002. While protein levels expression were only increased modestly (2 fold) by transfection of an IRF7 expressing vector (Fig. 5A) HIV-1 replication was markedly upregulated (14 fold) indicating enhanced viral replication when IRF7 is present in sufficient quantity (Fig. 5B). This occurred despite IFNα/β expression in the presence or absence of IRF7 overexpression (data not shown). This is the first demonstration that links IRF7 to an increased HIV-1 replication.

## Discussion

Our investigation demonstrated the capability of HIV-1 infection to alter critical early events involved in appropriate induction of the IFN response. This ability emphasizes the importance of the IFN signaling pathways in controlling HIV-1 replication and suggests that HIV-1 has evolved mechanisms to compromise this innate host immune response to favor its propagation. We present evidence that *TRAF6,* important for transcriptional regulation of the interferon pathway, is affected early in the process of HIV-1 infection of primary human macrophages. Knockdown of the antiviral factor *TRAF6* resulted in a significant augmentation in HIV-1 replication, as assessed by the expression of *TAT spliced* message.

TRAF6 has been shown to be important for control of replication of other viruses. Konno and collaborators reported that the absence of TRAF6 in *TRAF6^-/-^* mouse embryonic fibroblasts, (MEF cells) resulted in enhanced replication of RNA viruses such as Newcastle disease virus and encephalomyocarditis virus [23]. Similarly, TRAF6 has been shown to be critical for IFNα and β induction in response to vesicular stomatitis virus infection and intracellular double-stranded RNA, poly (I:C) [35]. All of these studies confirm that TRAF6 is induced as part of the normal innate immune response against viruses and our findings indicate it plays a similar role in curtailing HIV-1 replication. Interestingly, our results also suggest that HIV-1 has devised strategies to circumvent this inhibition. HIV-1 infection reduced TRAF6 gene expression with the resulting enhanced viral replication. Despite being upregulated by interferon and repressed by HIV-1 infection, *VISA*, another important mediator of innate immunity, did not have any impact on HIV-1 replication in our study. Instead, HIV-1 replication was similar to that of the control. There may be compensatory mechanisms circumventing the ablation or reduction of *VISA* expression during HIV-1 infection.

TRAF6 is implicated in the activation of the transcription factors IRF3 and IRF7 [22], [36]. TRAF6 polyubiquitinates IRF7, a post-translational modification necessary for the IFN production as demonstrated in fibroblasts [22], [36]. Both transcription factors are key regulators of IFN production [37]–[44] and have essential roles in the activation of antiviral immunity [44]–[46]. Certain viruses have evolved mechanisms to overcome their effect such as the Thogoto virus ML protein that reportedly inhibits the activation of IRF7 [47]. In addition, vesicular stomatitis virus was more efficient at infecting fibroblasts when IRF7 was ablated [48]. In contrast, IRF7 increased human papillomavirus (HPV) type 8 late promoter activity via direct binding to viral DNA and IRF3 induced strong HPV8 suppression in primary keratinocytes. This is consistent with our study where IRF7 promoted HIV-1 replication [49]. The knockdown of TRAF6 induced the expression of IRF7 but decreased the expression of IRF3 at both the gene and protein levels.

Overexpression of IRF7 resulted in a significant increase in HIV-1 replication in primary human macrophages. Moreover, inhibition of IRF7 brought about a concomitant decrease of viral output. Our results show that when TRAF6 is reduced, IRF7 is overproduced and contributes to enhanced viral replication. Recently, a study by Smith and collaborators identified host genes expressed in inguinal lymph nodes that were associated determinants of HIV-1 viral load [18]. Moreover, in primate models, higher levels of type 1 interferon characterize the pathogenic state [19]. Consistent with our results with IRF7, these analyses reflect an antiviral host response mediated by the interferon pathway that is associated with higher viral load rather than inhibition of HIV-1 replication, suggesting that HIV-1 subverts the innate immune response to its own benefits. IRF7 could contribute to enhanced HIV-1 replication by several potential, non-exclusive mechanisms. IRF7 could facilitate Long Terminal Repeat (LTR) driven expression of the virus and/or favor transcriptional activation of cellular genes that could contribute to increased viral output. However, on its own IRF7 is not capable of enhancing the activity of a LTR-luciferase reporter in primary human macrophages (Figure S1). Thus, if IRF7 modulates the HIV-1 LTR, it may require interaction with another protein. In opposition, IRF1, a positive control in this setting, could readily enhance LTR-driven replication. Presumably, IRF7 could facilitate the translocation and activity of NFkB resulting in the promotion of HIV-1 replication since NFkB is a potent activator of LTR-driven expression of the virus. Although demonstrated in Jurkat T cells, IRF7 could, in a situation analogous to IRF1, interact with NFkB to promote this LTR-driven transcription [50], [51]. Preliminary chromatin immunoprecipitation experiments (ChIP on chip monitoring 19,000 genes) using HIV-infected primary macrophages indicate enrichment for the promoter regions of three genes that stimulate the NFkB pathway, coactivator-associated arginine methyl transferase 1 (CARM1), B-cell CLL/lymphoma 10 (BCL10) and solute carrier family 20 member (SLC20A1) [52]–[54]. These interesting candidates warrant additional investigation to determine if they promote HIV-1 replication.

Alternatively, the increase in IRF7 expression together with the reduction in IRF3 expression might favour the formation of IRF7 homodimers instead of IRF3 homodimers or IRF3/IRF7 heterodimers. IRF3 and IRF7 require dimerization among other post-translational modifications to act as transcription factors. This shift in the transcription factor complex composition would induce the activation of a distinct set of genes some of which could contribute to HIV-1 replication. There is evidence in paramyxovirus infection of lymphocyte B-cells that the relative ratio of IRF3/IRF7 contributed to differential expression of IFNα2-related genes [55].

In conclusion, we demonstrated that TRAF6 is an important factor involved in the replication of HIV-1 in primary human macrophages. HIV-1 infection downregulated TRAF6 expression. In the absence of TRAF6, IRF7 is overproduced and contributes to enhanced HIV-1 replication. It is important to elucidate how HIV-1 interferes with this innate immunity in order to favour its own replication.

## Methods

### Cells

Monocytes were recovered from whole blood of healthy human donors by negative selection with RosetteSep™ human monocyte enrichment cocktail (Stem Cell Technologies Inc, Vancouver, BC), according to the manufacturer's instructions. A total of 15 independent donors were utilized for the experiments described in this manuscript. Monocytes to be differentiated into macrophages were plated into T25 flasks in RPMI 1640 supplemented with 10% human serum (Wisent Inc., Québec, QC) for oligonucleotide array experiments and subsequent validation by real-time quantitative RT-PCR **(**qRT-PCR). qRT-PCR quantification, knockdown assays and western blot assays used monocytes plated in RPM1 1640 with 10% fetal bovine serum (Invitrogen Canada, Burlington, ON) and 25 ng/ml of monocyte/macrophage colony-stimulating factor (M-CSF) (Genscript Corp, Piscataway, NJ) for differentiation into macrophages. Cell-surface expression of CD14 was characterized by using fluorescein isothiocyanate (FITC)-conjugated mAb (clone MEM-18) (Cedarlane Laboratories Limited, Burlington, ON) after allowing monocytes to differentiate for 5 days. By this criterion, the cells were >97% monocytes differentiated macrophages (MDMs) as assessed by flow-cytometry evaluation by using EPICS XL (Beckman Coulter, Fullerton, CA) (data not shown).

### Production of virus stocks

Virus stocks were produced by the transfection of 293T cells using the calcium phosphate co-precipitation method [56]. The infectious molecular clone used in this study was pNL4-3BaL*env.* The pNL4-3BaL*env* vector was generated by replacing the *env* gene of the T-tropic HIV-1 strain, NL4-3, with that of the macrophage-tropic HIV-1 BaL strain, thus resulting in an infectious molecular clone with macrophages-tropic properties [57] (pNL4-3BaL*env* was kindly provided by M. J. Tremblay, Laval University, Québec, QC). Supernatants from transfected cells were clarified by filtration through 0.22 micron cellulose acetate syringe filter and ultracentrifuged. Viruses were purified with the OptiPrep velocity gradient method (Axis-Shield PoC, Oslo, Norway). This methodology precludes having secreted products such as cytokines in the viral preparations. The 50% tissue culture infectious dose (TCID 50) of HIV-1 stock was calculated by using the nonparametric methods of Spearman-Kärber [58]. TZM-BL cell line was used to standardize every viral stock used in this study.

### Treatment and infection of macrophages

After allowing monocytes to differentiate for 5 days, purified primary macrophages were treated 18 hours with 1000 IU/ml of IFNα2 (PBL Biomedical Laboratories, Piscataway, NJ), which represents the lowest concentration achieving maximum inhibition of HIV-1 NL4-3BaL*env* strain replication in these cells [3]. Pre-treated macrophages (18 hours) were infected with the NL4-3BaL*env* strain of HIV-1 at a MOI of 0.002 (physiological dose). Uninfected and untreated cells were used as controls. Aliquots of cells (3×10^6^ cells) were taken at 2, 4, 8, and 24 hours after infection, lysed in 1 ml of TRIzol (Invitrogen Canada, Burlington, ON) and stored at −80°C.

### RNA isolation, labeling, and array hybridization

Total RNA from the same donor was isolated for each condition and time point using the TRIzol method according to the manufacturer's instructions (Invitrogen Canada, Burlington, ON) and then digested with deoxyribonuclease to remove any contaminating genomic DNA (Turbo DNA-free, Ambion, Applied Biosystems Canada, Streetsville, ON). RNA quality and quantity was assessed using an Agilent Technologies 2100 bioanalyzer and RNA 6000 Nano LabChip kit (Agilent Technologies Canada, Mississauga, ON). RNA integrity numbers (RIN), which estimate the integrity of total RNA samples, ranged from intact (RIN 10) to degraded (RIN 2). RIN were above eight for all samples in our experiments. Total RNA (100 ng) was converted to complementary DNA (cDNA), which was amplified and transcribed to produce biotinylated cRNA using the Two-Cycle cDNA synthesis kit (Affymetrix, Santa Clara, CA). Fragmented cRNA (15 µg) were hybridized to Affymetrix Human Genome U133 Plus 2.0 arrays (Affymetrix, Santa Clara, CA) for 16 h at 45°C with constant rotation at 60 rpm. The arrays were washed and stained with streptavidin-phycoerythrin (Molecular Probes, Eugene, OR) and biotinylated goat anti-streptavidin (Vector Laboratories, Burlingame, CA) using the Affymetrix Fluidics Station 450 (protocol EukGE-WS2v5_450), then read using the Affymetrix GeneChip Scanner 3000.

### Oligonucleotide array gene expression analysis

GeneChip Robust Multi-array Average (GCRMA) was the procedure used to normalize the data obtained from the oligonucleotide array analysis [59]. Identification of the most differentially expressed genes between IFNα2 pre-treated macrophages infected or not with HIV-1 was done using the short time series analysis framework of Shah and Corbeil [33]. This framework generalizes the Hilbert-Schmidt Independence Criterion (HSIC) based framework of Song and collaborators [60] to the short time-series setting by utilizing tensor analysis techniques resulting in a generic analysis tool that allows both identification of most differentially expressed genes and patterns of interest in gene behavior (such as upregulation by IFNα2 with subsequent suppression by HIV-1). We incorporated a linear kernel in the framework, and obtain a ranking criterion, which is analogous to the classical mean-difference based criterion for static gene expression data [33], [61]. The criterion is based on averaging the differences between consecutive measurements scaled by their respective means, over all the time points. The framework also allows identification of highly non-monotonic variations in gene behavior. The most differentially expressed genes were then extracted using an empirically determined threshold over the ranking scores. These genes were then analyzed with respect to their functionalities and ontological classification using the DAVID analysis software to determine significant functionalities associated with HIV-1 infection after IFNα2 treatment of the cells [34]. Furthermore, the top 500 differentially expressed genes between IFNα2 macrophages that were either infected with HIV-1 or non-infected were analyzed to determine which genes had an association with the keyword interferon using Chilibot software [62].

### qRT-PCR examination of gene expression

cDNA from independent donor cultures was generated from 200 ng of total RNA using a random primer hexamer following the instructions for Superscript II (Invitrogen Canada, Burlington, ON). Primers were designed using Primer Express 2.0 (Applied Biosystems Canada, Streetsville, ON) and their sequences are presented in Table 4. Amplicons were detected in most cases using the Amplifluor UniPrimer amplification and detection system (Chemicon International, Temecula, CA) except for *TAT spliced* message, a marker of HIV replication, which was detected using the TaqMan system (Applied Biosystems Canada, Streetsville, ON), which allows for greater sensitivity and can detect infection as early as 2 hours post-inoculation. Forward primers used in the Amplifluor UniPrimer system contained an additional 5′ Z sequence (ACTGAACCTGACCGTACA) that is not included in Table 4. Equal amounts of cDNA (20 ng) were run in triplicate and amplified using the Amplifluor Uniprimer in a 15 µl reaction containing 7.5 µl of 2X Universal PCR Master Mix (Applied Biosystems Canada, Streetsville, ON), 10 nM of Z-tailed forward primer, 100 nM of reverse primer, 100 nM of Amplifluor Uniprimer fluorescein probe (Chemicon International, Temecula, CA) and 5 µl of DNA target [63]. Moreover, no-template controls were used. The mixture was incubated at 50°C for 2 min, at 95°C for 4 min, then cycled at 95°C for 15 sec and at 55°C for 40 sec, 55 times using the Applied Biosystems 7900HT Sequence Detection System. The amplifications using the Taqman system were run in a 15 µl reaction containing 7.5 µl of 2X Universal PCR Master Mix, 200 nM of forward primer, 200 nM of reverse primer, 250 nM of Taqman probe and 5 µl of DNA target. The mixture was incubated at 50°C for 2 min, at 95°C for 10 min, then cycled 40 times at 95°C for 15 sec and at 60°C for 1 min. Amplification efficiencies were validated and normalized to ribosomal 18 S and quantity of target gene (arbitrary units) was calculated according to a standard curve. The standard curve consists of different dilution of a RNA sample that generates data points in the linear portion of the PCR amplification.

**Table 4:** Sequence of Oligonucleotide Primers used in Real-Time qRT-PCR Gene Expression Analysis.

Primer^a^	Nucleotide Sequence
TRAF6-F	AAGGGATGCAGGTCACAAATGT
TRAF6-R	TTTTCCAGCAGTATTTCATTGTCAA
VISA-F	ACTTCATTGCGGCACTGAGG
VISA-R	CTTCGTCCGCGAGATCAACT
IRF7-F	CGACATCGAGTGCTTCCTTATG
IRF7-R	ACTGGGTTCTAGGCGGGC
IRF3-F	TCTGATACCCAGGAAGACATTCTG
IRF3-R	CAACACCATGTTACCCAGTAACTCAT
TAT Spliced-F^b^	CCTAAAACTGCTTGTACCAATTGC
TAT Spliced-R^b^	GGAGGTGGGTTGCTTTGATAGAGA
TAT Spliced-probe^b^	AAAGCCTTAGGCATCTC

a F (forward) primer sequence is in 5′-3′ orientation, R (reverse) primer sequence is reverse complemented.

b TAT Spliced was detected using Taqman.

### Enzyme-linked immunosorbent assay (ELISA)

Macrophages were infected with HIV-1 at a MOI of 0.002 for 2 hours at 37°C. Next, the virus-cell mixture was washed with PBS to remove unbound virus. The p24 content was determined using a sensitive in-house double-antibody sandwich ELISA specific for the viral p24 protein. In this test, the 183-H12-5C and 31-90-25 antibodies are used in combination to quantify p24 levels. Virus production was estimated by measuring p24 levels in cell free culture supernatants.

### Knockdown assays

Macrophages from healthy donors were transfected with pre-designed siRNA SMARTpool for *TRAF6* (M-004712-00; Dharmacon, Lafayette, CO), *VISA* (L-024237-00; Dharmacon, Lafayette, CO) or *IRF7* (L-011810-00; Dharmacon, Lafayette, CO) with the Oligofectamine Transfection Reagent according to the manufacturer's instructions (Invitrogen Canada, Burlington, ON). SMARTpool technology combines four siRNAs that target different mRNA regions. Silencer Negative control siRNAs (AM4635; Ambion, Applied Biosystems Canada, Streetsville, ON), used as control do not target any human gene product. The best gene expression inhibition levels of 65%, 61% and 55% were obtained for *TRAF6, VISA* and *IRF7*, respectively, 48 hours post-transfection (see Figure S2 for TRAF6 results). The transfected macrophages were then infected with the NL4-3BaL*env* strain of HIV-1 at an MOI of 0.002 for 24 hours. In the case of IFNα2 pre-treatment, 30 hours post-transfection the macrophages were treated for 18 hours before the infection.

### Protein assays

Immunoblot assays were performed on total cell lysates of independent donors. Proteins were isolated for each condition using total extract buffer (Tris-base pH 6.8, SDS 20%, mercaptoethanol and glycerol). Cell extracts (30 µg per lane) were resolved by SDS-PAGE on 4–20% gels (BIO-RAD Laboratories Canada Ltd, Mississauga, ON) and transferred on PVDF membranes. The indicated antibodies against IRF7 (H-246; Santa Cruz Biotechnology, Santa Cruz, CA), IRF3 (SL-12.1; BD-Pharmingen, Oakville, ON), TRAF6 (H-274; Santa Cruz Biotechnology, Santa Cruz, CA) and β-actin (A-13; SIGMA, Saint-Louis, MO) were visualized by alkaline phosphatase-based enhanced chemiluminescence. The densitometry of the bands was compared after normalization with β-actin.

### Overexpression assays

Macrophages (15×10^6^ cells) from healthy donors were transfected with 9 µg of pcDNA3-IRF7-myc (Kindly provided by J. Hiscott, McGill University, Montréal, QC) or pcDNA3 (empty vector control) with the Lipofectamine Transfection Reagent according to the manufacturer's instructions (Invitrogen Canada, Burlington, ON). We obtained the best gene overexpression after 24 hours of transfection, after which, the transfected macrophages were infected with the NL4-3BaL*env* strain of HIV-1 at a MOI of 0.002 for 24 hours.

### Microarray data accession number

Microarray results have been deposited in Gene Expression Omnibus database under accession number GSE30536.

### Statistical analysis

Analysis was performed by paired *t*-test to compare two population means in the case of two samples that are correlated (same donor) or one sample *t*-test to compare the sample mean to the population mean (control). P values of less than 0.05 were considered to be statistically significant.

### Ethics statement

Comité d'éthique de la recherche du CHUQ approved the study and written informed consent was provided by study participants.

## Supporting Information

Figure S1 **Transactivation of the HIV-1 LTR by IRF1, IRF3 and IRF7.** Expression vector for IRFs and pBlue_5′_LTR_LUC were co-transfected in primary macrophages. Luciferase activity (RLU) was measured at 24 h post-transfection. Results are the mean of two separate experiments.
(TIF)

Figure S2 **Western blot for TRAF6 24 hours post-infection of macrophages knockdown with 50, 100, 200 and 500 nM of** ***TRAF6*** **siRNAs.** β-actin was used as a normalizer for input. 200 nM of siRNA were used since a better percentage of inhibition in *TRAF6* knockdown experiments were obtained at this concentration with respect to the protein level (67%).
(TIF)
