# Supplementary figures and images for: TRAF6 and IRF7 Control HIV Replication in Macrophages
Source: PLoS One. 2011 Nov 28;6(11):e28125. doi: 10.1371/journal.pone.0028125 (PMC3225375; doi:10.1371/journal.pone.0028125)

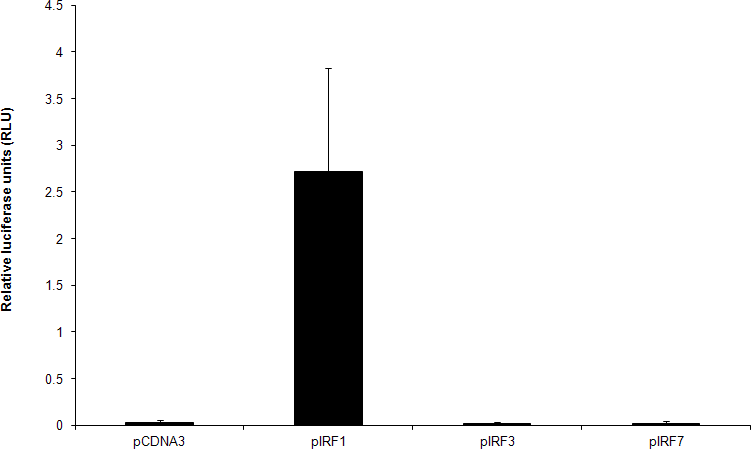

Supplement: Figure S1 — Transactivation of the HIV-1 LTR by IRF1, IRF3 and IRF7. Expression vector for IRFs and pBlue_5′_LTR_LUC were co-transfected in primary macrophages. Luciferase activity (RLU) was measured at 24 h post-transfection. Results are the mean of two separate experiments. (TIF) [file pone.0028125.s001.tif]

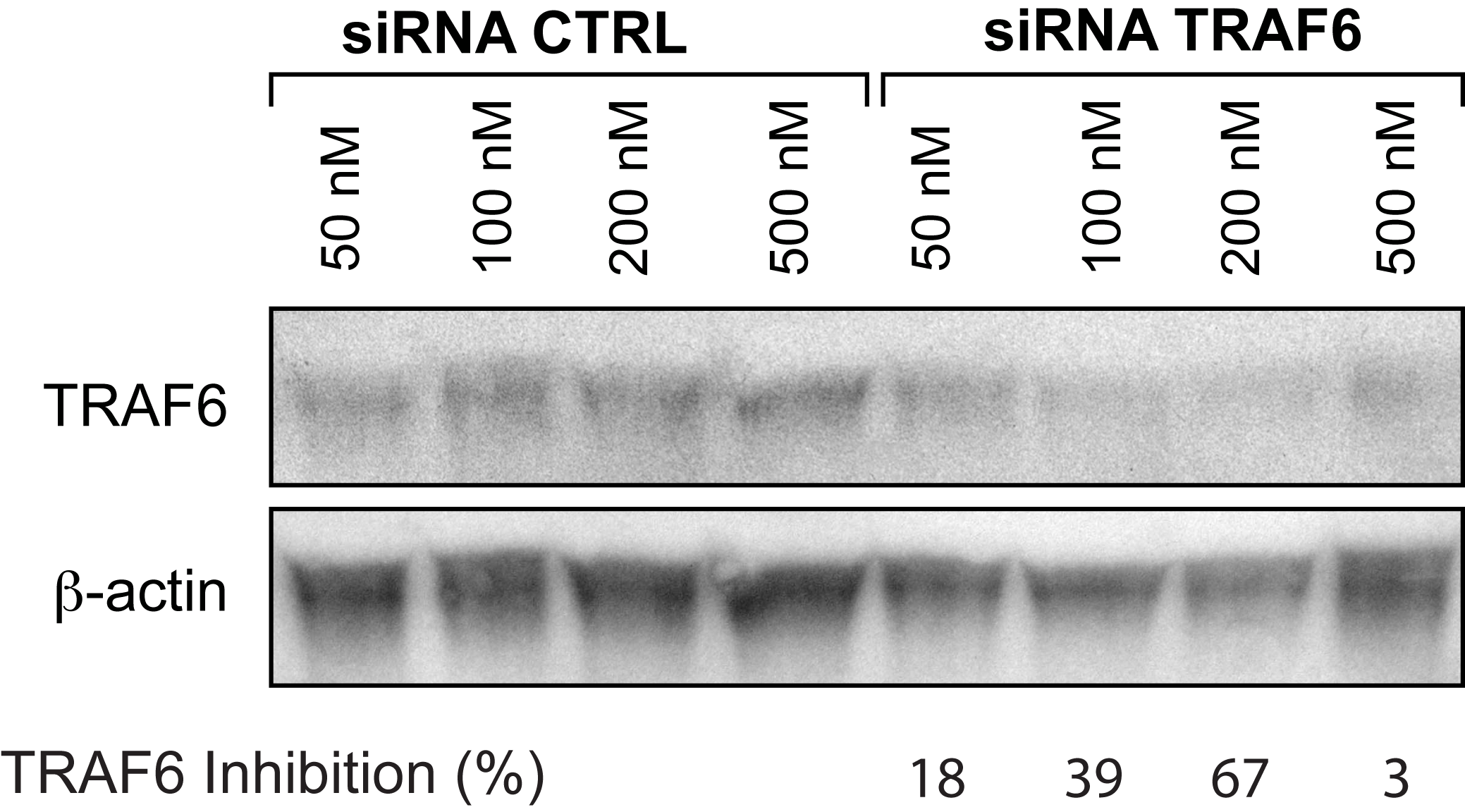

Supplement: Figure S2 — Western blot for TRAF6 24 hours post-infection of macrophages knockdown with 50, 100, 200 and 500 nM of TRAF6 siRNAs. β-actin was used as a normalizer for input. 200 nM of siRNA were used since a better percentage of inhibition in TRAF6 knockdown experiments were obtained at this concentration with respect to the protein level (67%). (TIF) [file pone.0028125.s002.tif]
